# Case of Accidental Trauma Resulting in Condylar Fractures in 3-Month-Old

**DOI:** 10.1155/2023/7611475

**Published:** 2023-10-04

**Authors:** Maggie M. Mouzourakis, Sarah S. Seo, Resmiye Oral

**Affiliations:** ^1^Section of Otolaryngology, Department of Surgery, Dartmouth-Hitchcock Medical Center, Lebanon NH, USA; ^2^Surgery, Geisel School of Medicine, Lebanon NH, USA; ^3^Child Advocacy and Protection Program, Dartmouth-Hitchcock Medical Center, Lebanon NH, USA; ^4^Pediatrics, Geisel School of Medicine, Lebanon NH, USA

## Abstract

While mandibular fractures represent the most common craniofacial injury in the pediatric population, craniofacial fractures in infants are rare. Diagnosis is challenging with nonspecific presenting symptoms and often limited radiographic imaging. We report a case of nondisplaced mandibular condylar head fractures in a 3-month-old patient as a result of a fall with impact onto the chin, with associated sublingual hematoma (Coleman's sign). Although the initially observed sole finding of sublingual hematoma raised concern for child physical abuse, detailed history, oral exam, and multidisciplinary review of radiologic records by pediatrics, otolaryngology, and child protection teams established the accidental trauma diagnosis. The patient was managed conservatively with normal resumption of feeding. Detailed history and multidisciplinary approach to the management of pediatric facial trauma are important in establishing diagnoses and management.

## 1. Introduction

Oral injuries in young infants are often observed in the context of child physical abuse. Oral hematomas and frenulum injuries in the absence of a plausible accidental mechanism of trauma specifically raise concern for child physical abuse [1, 2]. One of the differential diagnostic possibilities especially for sublingual hematoma is what Dr. Frank Coleman coined as “Coleman's sign” [3]. He commented in 1912 that mandibular fractures could generate shear forces to the mandibular periosteum or fascia, resulting in extravasation of blood sublingually, which he reported to be pathognomonic for mandibular fractures.

Mandibular condylar fractures (MCF) represent one of the most common facial fractures in the pediatric population [4]. Injuries frequently result from a force translating from the anterior mandibular site of injury posteriorly to the condylar heads [5–7]. Associated symptoms include skin bruising over the anterior mandible, trismus, or changes to dentition. Injuries of the bilateral condyles can be associated with a symphyseal/parasymphyseal mandibular injury [6].

While accidental MCF are common in the pediatric population, it is rare to see this injury in children under the age of two. Previous work has discussed that the work-up for accidental vs nonaccidental trauma can be challenging [8]. In this case report, we discuss the history of trauma and accidental versus nonaccidental trauma (NAT) differential diagnostic process in a 3-month-old child who presented with sublingual hematoma, a very small bruise on the chin after a trivial fall with subsequent diagnosis of bilateral MCF. Parental consent was obtained to publish this case.

## 2. Case Report

This is a three-month-old female who was born at full term initially presenting for challenges with feeding and colic. Father had noted that the evening prior, she rolled off the couch and landed on her chin. No bruising was noted, and she continued to be interactive without changes in behavior at the time of the injury. Starting the next day, she refused any attempts at breastfeeding. Parents noted that with crying, she would tug at her right ear. Due to limited intake over a 24 hr period, she was brought to the emergency room for initial evaluation. No other problems were noted on the review of systems.

**Figure 1:**
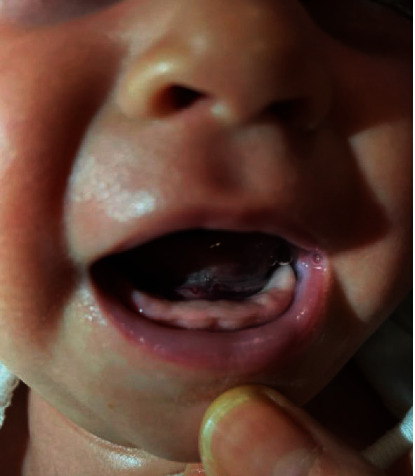
Sublingual hematoma. Evidence of bruising and coalescent blood in the sublingual space without mucosal disruption, suggestive of Coleman's sign.

Physical exam revealed a sublingual hematoma, without evidence of lacerations or other mucosa injuries (Figure 1). The rest of the physical exam was within normal limits. Based on the initial presentation of sublingual hematoma without plausible intraoral trauma or external bruising, child abuse work-up was initiated.

**Figure 2:**
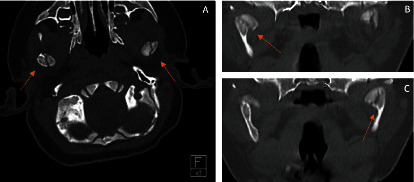
Initial CT scan in axial view (A). Coronal view of right condylar fracture (B). Coronal view of left condylar fracture (C).

A skeletal survey did not reveal any abnormalities, or evidence of anterior mandibular fractures. The head CT showed bilateral high medial nondisplaced MCF (Figure 2). A team of multiple radiologists as well as the oral maxillofacial surgery team reviewed the images and compared them to normal head CT scans of infants to finalize the CT read. A retinal exam revealed no retinal bleeding.

The patient was admitted to the hospital from the emergency room to complete the rest of the work-up, which included social work consultation and filing a report with the child protective services. Social work and child abuse consults did not reveal any risk factors for child abuse and neglect, and the history of trauma remained consistent. Laboratory studies other than the head CT findings remained within normal limits.

**Figure 3:**
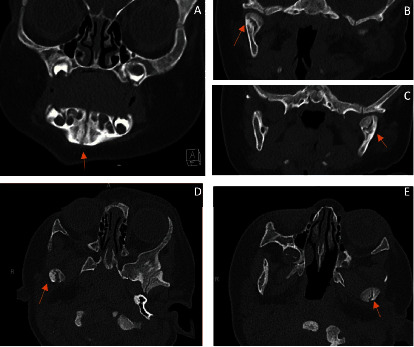
Follow-up CT scan 2 weeks after in coronal view, with no evidence of symphyseal or parasymphyseal fracture, mandibular symphysis visualized (A). Coronal view of right condylar fracture (B). Coronal view of left condylar fracture (C). Axial view of right condylar fracture (D). Axial view of left condylar fracture (E).

A nasogastric tube was inserted at the start of her hospital admission to augment nutrition. Over the next 24 hours, she began feeding again by bottle with a reduced flow Dr. Brown nipple. A small bruise was noted to have developed along her anterior chin by the next day (hospital day 1). The nasogastric tube was removed, and the patient was discharged to parents' custody (hospital day 2). At 2 weeks, an outpatient repeat facial CT scan was done to rule out any possibility of mandibular symphyseal or parasymphyseal injury. The facial CT (Figure 3) demonstrated healing nondisplaced MCF without evidence of other mandibular injury. By the oral maxillofacial surgery follow-up clinic visit 3 weeks later, she was back to breastfeeding normally, with no evidence of discomfort or pain. On exam, the sublingual swelling and hematoma had resolved. She had normal mobility and mouth opening, with no evidence of trismus. Plans were made to continue to progress her diet as planned to table foods at 6 months of age and schedule an orthodontic evaluation at 7 years of age.

## 3. Discussion

Pediatric craniofacial injuries make up only 10% of total reported craniofacial injuries [9]. Previous studies have reported MCF to be common in children, although it is relatively uncommon to see these injuries in infants [10–12]. Perhaps, owing to the elasticity of bone as well as limited mobility prior to 6 months of age, MCF have been very rarely reported in the infant age group [8, 13]. Recent limited cases of MCF in infants have been included as part of case series of all pediatric patients with MCF [4, 8, 14].

Signs and symptoms of mandibular fractures in infants can be nonspecific and require a detailed parental history prior to diagnosis. In this case report, the primary presentation was the failure to breastfeed. Upon seeking medical care, an unexplained sublingual hematoma was discovered which raised concern for child physical abuse. The history provided by the parents and multidisciplinary approach became crucial in differentiating between accidental versus nonaccidental injury.

The majority of mandibular fractures in infants occur in an accidental context [4, 15]. However, nonaccidental trauma is also a leading cause of craniofacial injury in infancy [16, 17]. Sublingual hematoma, which was the first sign of injury found in this patient, is a common finding in NAT as a result of forceful feeding and direct impact trauma. Clyde et al. described bruising patterns in children and found that characteristic bruising matching TEN-4-FACESp (torso, ear, neck (TEN), under 4 months (4) frenulum, angle of jaw, cheeks (fleshy), eyelids, subconjunctival (FACES), and patterned (p)) was highly associated with NAT [18]. Distinguishing NAT from accidental MCF can be challenging given similar presentations. Since MCF often present with a sublingual hematoma, Coleman's sign, a sign of indirect shear forces, accidental MCF should be considered in differential diagnosis, especially in an infant of this age [1–3]. In a recent study, 5/8 mandible fractures in children under the age of 24 months were diagnosed as accidental, and 1/6 was diagnosed as child abuse [8]. Of note, in a survey of pediatric patients with accidental trauma and NAT, NAT was more commonly associated with patients of younger age, with multiple fracture sites and with MCF [17]. Thus, both inflicted and accidental MCF should be kept in mind until all information is available to establish an accurate differential diagnosis.

Careful oral examination should include mucosal injuries, sublingual hematoma, and other signs of forceful feeding to distinguish Coleman's sign from other causes of sublingual trauma. In this case report, the patient had extravasation of blood in the sublingual space without overt mucosal swelling, frenulum or mucosal tears, and other injury. The CT scan later revealed bilateral MCF, thus allowing the attribution of the sublingual hematoma to the shear forces involved in the mechanism of the fractures.

The multidisciplinary team consisting of oral maxillofacial surgery, radiology, and child abuse pediatrics reviewed the literature and compared the CT scans in this case to scans of a normal, same-age patient to finalize the diagnosis of bilateral nondisplaced medial mandibular condylar head fractures. Additionally, a hospital-based multidisciplinary team meeting including child protection services was held to arrive at a consensus decision that the injuries were consistent with an accidental impact trauma to the chin.

Mandibular condylar fractures are typically characterized by location, displacement, laterality, and injury to surrounding structures. The Lindahl and Strasbourg Osteosynthesis classifications subdivide MCF by height/location, displacement or fissuring, and location in the glenoid fossa [19, 20]. The fractures in this patient were high, medial, within the capsule, and nondisplaced, in the glenoid fossa. Most studies show that conservative therapy is appropriate for the management of MCF in the pediatric population [4, 6, 21]. The mandible develops from Meckel's cartilage of the first pharyngeal pouch, and much of the growth occurs in the first year of life from the cartilage at the condylar head [22, 23]. While there is always concern for growth anomalies arising from injury to the condyle, in a study involving long-term follow-up of 88 children treated at Hospital for Sick Children, most patients under the age of 2 managed conservatively did not require further later orthognathic intervention compared to older counterparts [24]. Any fixation or open treatments for mandibular fractures in the growing mandible would risk further disturbing growth, contributing to why most cases are treated conservatively [11, 12]. For children who develop TMJ ankylosis, further TMJ intervention is usually reserved for after age 6, when they are able to participate in rehabilitation therapy [24]. For this patient, we opted for close follow-up in the first 3 months, followed by orthodontic evaluation at age 7. She was also established with a local orthognathic surgeon for any future concerns regarding TMJ ankylosis or mandibular development.

## 4. Conclusion

We report a case of nondisplaced mandibular condylar head fractures in a 3-month-old patient as a result of a fall from height, with associated sublingual hematoma (Coleman's sign). Detailed history, oral exam and review of radiologic records, and a hospital-based multidisciplinary/interagency team meeting were important in establishing the accidental nature of the injuries. The patient was managed conservatively for the MCF and experienced normal recovery of function. Infantile craniofacial injuries are rare and we hope this case report expands upon a growing body of literature on this topic including differential diagnosis of accidental versus inflicted sublingual hematoma.

## Data Availability

The data used to support the findings of this study are available from the corresponding author upon request.
